# Role of ssDNA as a Noninvasive Indicator for the Diagnosis and Prognosis of Hepatocellular Carcinoma: An Exploratory Study

**DOI:** 10.1155/2021/9958909

**Published:** 2021-08-05

**Authors:** Qi Zhao, Yiqiu Xu, Dandan Yuan, Junjun Yang, Ying Wang, Guorong Shen, Xuewen Huang

**Affiliations:** ^1^Department of Clinical Laboratory, The Affiliated Wuxi People's Hospital of Nanjing Medical University, 299 Qingyang Road, Jiangsu Wuxi 214023, China; ^2^Department of Clinical Laboratory, The Affiliated Wuxi No. 2 People's Hospital of Nanjing Medical University, 68 Zhongshan Road, Jiangsu Wuxi 214002, China; ^3^Department of Infection Management, Suzhou Hospital Affiliated Nanjing Medical University, 68 Guangji Road, Jiangsu Suzhou 215008, China; ^4^Department of Clinical Laboratory Medicine, Suzhou Ninth Hospital Affiliated to Soochow University, 2666 Ludang Road, Jiangsu Suzhou 215200, China; ^5^Research & Development, Jiangsu Yuan Biotechnology Co., Ltd, 333 Xingpu Road, Jiangsu Suzhou 215000, China

## Abstract

**Methods:**

This prospective study enrolled 102 patients with newly diagnosed HCC, 21 with cirrhosis, 20 with chronic hepatitis, 284 with nonliver diseases, and 45 healthy individuals at the Affiliated Wuxi No. 2 People's Hospital of Nanjing Medical University (May-October 2018). ssDNA was extracted using magnetic beads and quantified using the Qubit ssDNA assay. ssDNA levels were compared among the disease groups and in HCC vs. non-HCC. Receiver operating characteristic (ROC) curves were used to determine the diagnostic value of ssDNA. In patients with resectable HCC, ssDNA and *α*-fetoprotein (AFP) levels were measured during follow-up and compared with HCC recurrence detected by imaging.

**Results:**

The median ssDNA levels were higher in HCC than in healthy individuals, cirrhosis, and chronic hepatitis (median, 23.20 vs. 9.36, 9.64, and 9.76 ng/*μ*L, respectively, *P* < 0.001). ssDNA levels in HCC were higher than those in cirrhosis and chronic hepatitis (both *P* < 0.001); there were no differences in ssDNA levels between healthy controls and patients with cirrhosis (*P* = 0.15) or chronic liver disease (*P* = 0.39). The area under the curve of ssDNA for HCC diagnosis was 0.909 (95% CI: 0.879-0.933). The ssDNA levels decreased by 3.19-fold (*P* < 0.001) after HCC radical resection. In six patients, the ssDNA levels increased about 3-6 months before a recurrence was detected by AFP and imaging.

**Conclusions:**

ssDNA might be a noninvasive indicator for HCC diagnosis and prognosis. ssDNA could eventually be complementary to AFP levels and imaging, but confirmatory studies are necessary.

## 1. Introduction

Hepatocellular carcinoma (HCC) is the fifth most common cancer and the third more important cause of cancer-related mortality worldwide [1]. A major etiology of HCC is chronic hepatitis B and C, which are endemic in countries like China, resulting in a high incidence of HCC [2], with 40.0 per 100,000 males and 15.3 per 100,000 females [3]. HCC develops due to hepatic injury and/or inflammation that leads to fibrosis and cirrhosis, abnormal hepatocyte regeneration, and the formation of preneoplastic lesions [4, 5]. Otherwise, the specific pathogenesis depends upon the specific etiology. Hepatotropic viruses cause inflammation, cell injury, increased cell turnover, fibrosis, and cirrhosis [5]. Alcohol-related HCC is related to oxidative stress due to ethanol metabolism and inflammation [5]. Nonalcoholic steatohepatitis (NASH) is associated with oxidative stress, insulin resistance, adipocytokine functional disorder, and cell hyperplasia leading to carcinogenesis [5].

Alpha-fetoprotein (AFP) is a biomarker for HCC diagnosis and monitoring, and increased AFP levels can be detected in 39%-65% of HCC patients, but many patients with HCC have low AFP levels (i.e., AFP < 20 ng/mL) [6]. Therefore, there is a need for complementary biomarkers. The combination of AFP and other traditional tumor markers (TTMs) for the diagnosis of HCC is a possible strategy [7–9]. New biomarkers such as Golgi protein 73 (GP73) [10], glypican-3 (GPC-3) [11], and microRNAs [12–14] are under investigation, but low sensitivity and/or specificity for HCC limit their application in clinical practice [10–14].

Cell-free DNA (cfDNA) mostly comes from apoptotic and necrotic cells and contains the complete genetic information of their tissue of origin. cfDNA has been suggested as a dynamic real-time marker of HCC burden in patients with various treatments and as a possible means to detect tumor mutations [15]. Studies on cfDNA in HCC diagnosis focused on detecting tumor mutation and methylation information using various techniques, but these techniques are expensive, and the results are conflicting [16–20]. Previous studies suggested that the quantitative measurement of cfDNA might have diagnostic and prognostic values for HCC [21, 22], but the quantitative analysis of cfDNA does not provide information about the biological and molecular characteristics of HCC. Although the usefulness of cfDNA quantitative analysis in HCC is controversial, it has advantages such as being simple, fast, and inexpensive [23, 24].

When considering the high proportion of AFP-negative HCC [6] and the association between cfDNA and HCC [21, 22], cfDNA could be used to detect HCC among AFP-negative patients. cfDNA consists of double-stranded DNA (dsDNA) and single-stranded DNA (ssDNA). Previous studies on the quantitative changes of cfDNA focused on dsDNA. ssDNA is an earlier marker of replication stress than dsDNA [25–27]. In cells with high replication stress and uncontrolled replication (such as cancer cells), stalling or collapse of the replication fork due to uncoordinated enzymes leads to ssDNA formation and release by apoptotic cells [25, 26]. In addition, the plasma levels of ssDNA are much higher than that of dsDNA [23, 24].

Therefore, it could be hypothesized that the plasma ssDNA is a marker of HCC. This exploratory study is aimed at exploring the diagnostic and prognostic values of ssDNA for HCC.

## 2. Materials and Methods

### 2.1. Study Design and Patients

This prospective exploratory study enrolled patients with newly diagnosed HCC (*n* = 102), cirrhosis (*n* = 21), chronic hepatitis (*n* = 20), and nonliver conditions (*n* = 284) at the Affiliated Wuxi No. 2 People's Hospital of Nanjing Medical University between May and October 2018. They were identified based on symptoms, imaging findings, biopsy, TTMs including AFP and cancer antigen (CA) 12-5, and other serum markers [28–30]. A given patient was included only once at its first admission. The study was approved by the ethics committee of the Affiliated Wuxi No. 2 People's Hospital of Nanjing Medical University (#Y-25). The participants provided written informed consent before any study procedure.

Healthy individuals (*n* = 45) were enrolled when they visited the hospital for a routine checkup. The inclusion criteria were no tumor and liver markers above the upper limit of normal and normal physical examination (computed tomography (CT), upper gastrointestinal endoscopy, and abdominal ultrasound). The exclusion criteria were (1) <30 years of age, (2) previous therapies (major surgery, chemotherapy, endocrine therapy, or chronic treatment with any drug), (3) benign tumors, (4) chronic inflammatory disease (diabetes, cardiovascular diseases, or rheumatoid diseases), or (5) any autoimmune disease.

For the analysis of the ssDNA levels in liver conditions, the participants were grouped as HCC (*n* = 102), cirrhosis (*n* = 21), chronic hepatitis (*n* = 20), and healthy individuals (*n* = 45). For the diagnostic value analysis, the participants were grouped as the HCC (*n* = 102) and non-HCC (*n* = 325) groups. Resectable HCC was defined as any HCC that was judged to be resectable by surgeons specialized in oncological liver surgery according to the Child-Pugh classification [28, 30]. AFP-positive HCC was defined as patients with AFP > 20 ng/mL [31].

### 2.2. Blood Sampling

Fasting peripheral blood was sampled before any treatment using 4 mL EDTA vacuum tubes (BD Biosciences, Franklin Lakes, NJ, USA) and 4 mL gel procoagulation vacuum tubes (BD Biosciences, Franklin Lakes, NJ, USA). For patients with resectable HCC, blood was sampled on days 3, 14, 30, and 60 after surgery. The resected specimen was examined. The clinical or pathological staging of HCC was performed according to the tumor node metastasis (TNM) classification [32]. The tumor size was according to the Response Evaluation Criteria in Solid Tumor (RECIST) 1.1 [33]. Abdomen and pelvic CT was performed 60 days after surgery (before chemotherapy) and as the baseline for RECIST assessment.

For patients with HCC and ssDNA levels > 12.36 ng/*μ*L after radical resection, CT scan and blood sampling were performed every 3 months. Disease response was evaluated according to RECIST version 1.1: complete response (CR), partial response (PR), progressive disease (PD), and stable disease (SD).

### 2.3. ssDNA Quantification

The blood samples were processed within 1 h of the drawing. The plasma was obtained by centrifugation at 1900 × g for 10 min. The plasma was centrifuged again at 16,000 × g for 10 min. The collected plasma was stored at -80°C.

cfDNA extraction was performed using the Cell-free DNA Extraction Kit (Yuan Biotechnology, Jiangsu, China), based on the magnetic bead method and according to the manufacturer's instructions, within 2 h of sample thawing. Briefly, 1 mL of lysis adsorbent and 12.5 *μ*L of protease K were added to 0.5 mL of plasma and centrifuged at 1500 rpm and 60°C for 10 min. Then, 10 *μ*L of magnetic beads was added and centrifuged at 1500 rpm at room temperature for 10 min. The magnetic beads were captured on a magnetic frame. The cfDNA was eluted with 25 *μ*L of elution buffer B and stored at -20°C. Pooled plasma was used as quality control to monitor the changes in extraction efficiency.

The ssDNA levels were measured using 5 *μ*L of each sample and the Qubit ssDNA Assay Kit (Life Technologies, Carlsbad, CA, USA), according to the manufacturer's instructions. The quantification of ssDNA was performed at the 1 *μ*L mode using the Qubit 3.0 Fluorometer (Life Technologies, Carlsbad, CA, USA). Two replicates were performed for each sample. The coefficient of variation of the ssDNA concentration in the quality control had to be <5%.

### 2.4. Serum AFP, CA19-9, CA12-5, and CEA

The serum AFP, CA19-9, CA12-5, and carcinoembryonic antigen (CEA) levels were determined within 2 h using commercial test kits (Roche Diagnostics, Basel, Switzerland) on a Cobas e601 Analyzer (Roche Diagnostics, Basel, Switzerland), with an upper limit of normal of 9 ng/mL, 35 U/mL, 35 U/mL, and 5 ng/mL, respectively.

### 2.5. Statistical Analysis

The characteristics of the patients were presented according to the cut-off points of the BCLC classification [34]. The continuous variables were analyzed using the Mann–Whitney *U*-test or the Kruskal-Wallis test. Paired continuous variables were analyzed using the Wilcoxon test. Categorical variables were analyzed using the chi-squared test or the chi-squared test with correction for continuity. A receiver operating characteristic (ROC) curve analysis was used to analyze the diagnostic value of ssDNA. The value with the largest Youden index was selected as the optimal cut-off value. A value greater than or equal to the cut-off value was regarded as positive, and smaller values were regarded as negative. The relationships between the ssDNA levels and tumor size were analyzed by linear regression. *P* values < 0.05 were considered statistically significant. The area under the ROC curve (AUC) was reported using a two-sided 95% confidence interval (CI). The statistical analysis was performed using SPSS 16.0 (IBM, Armonk, NY, USA).

## 3. Results

### 3.1. Characteristics of the Subjects

Finally, 472 subjects were enrolled: 61 patients with AFP-negative HCC, 41 patients with AFP-positive HCC, 21 with cirrhosis, 20 with chronic hepatitis, 28 with coronary heart disease, 34 with autoimmune disease, 20 with diabetes, 53 with lung cancer, 43 with a colorectal tumor, 30 with a gastroduodenal tumor, 16 with head and neck tumor, five with genitourinary tumor, 19 with esophageal tumor, 11 with gynecologic tumor, nine with pancreatic cancer, and 16 with other tumors; 45 healthy individuals were also enrolled. Among the 102 patients with HCC, 37 were stage I, 42 were stage II, and 23 were stages III-IV. Among the 102 patients, 62 had resectable HCC (Table 1), 13 (12.8%) had lymph node metastases, nine (8.8%) had distant metastases, and 76 (74.5%) were hepatitis B virus- (HBV-) positive.

### 3.2. ssDNA Levels

**Figure 1:**
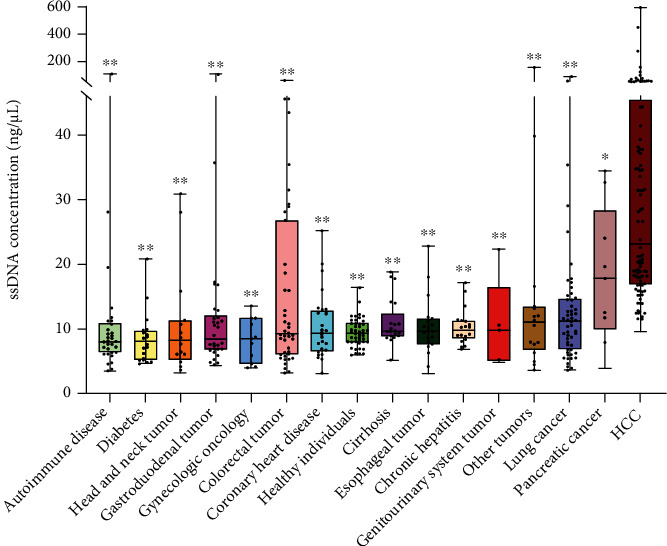
Comparison of single-stranded DNA (ssDNA) levels among patients with hepatocellular carcinoma (HCC), chronic liver diseases, nonliver conditions, and healthy individuals. The values (medians) are sorted from low to high. ^∗^*P* < 0.05 vs. HCC; ^∗∗^*P* < 0.01 vs. HCC.

**Figure 2:**
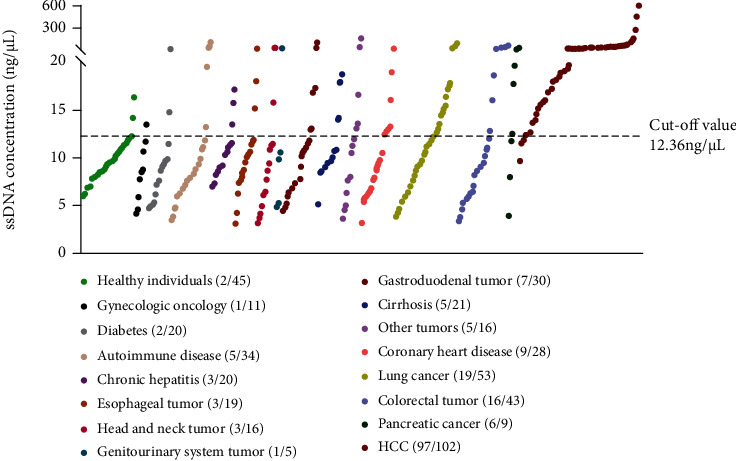
Comparison of the positive rate of single-stranded DNA (ssDNA) levels among patients with hepatocellular carcinoma (HCC), chronic liver diseases, nonliver conditions, and healthy individuals. The values (medians) are sorted from low to high.

As shown in Figures 1 and 2, the levels of ssDNA were higher in HCC (*n* = 102) compared with other diseases (all *P* < 0.01, except for pancreatic cancer, with *P* < 0.05). Among the three liver diseases, the ssDNA levels in HCC were higher than in cirrhosis (*n* = 21) and chronic hepatitis (*n* = 20) (both *P* < 0.001). There were no differences in ssDNA levels between healthy controls (*n* = 45) and patients with cirrhosis (*n* = 102) (*P* = 0.15) or chronic liver disease (*P* = 0.39) (Table 2).

### 3.3. ssDNA Levels in Patients with HCC

In HCC patients, there were no differences in ssDNA levels in different ages (*P* = 0.99), sex (*P* = 0.42), AFP-negative/positive HCC (*P* = 0.33), and cirrhosis HCC (*P* = 0.64) subgroups (Table 1). The ssDNA levels in unresectable HCC were higher than in resectable HCC (*P* = 0.02). There were no differences in ssDNA levels among subgroups of tumor size (*P* = 0.13) and no correlation between ssDNA levels and HCC size (*r* = 0.17, *P* = 0.10) (Supplementary Figure S1A). There were significant differences in ssDNA levels among patients with different TNM stages (*P* = 0.02) (Table 1). There was a rank correlation between ssDNA levels and TNM staging (rho = 0.274, 95% CI: 0.084-0.444, *P* = 0.005).

### 3.4. Diagnostic Value of ssDNA for HCC

**Figure 3:**
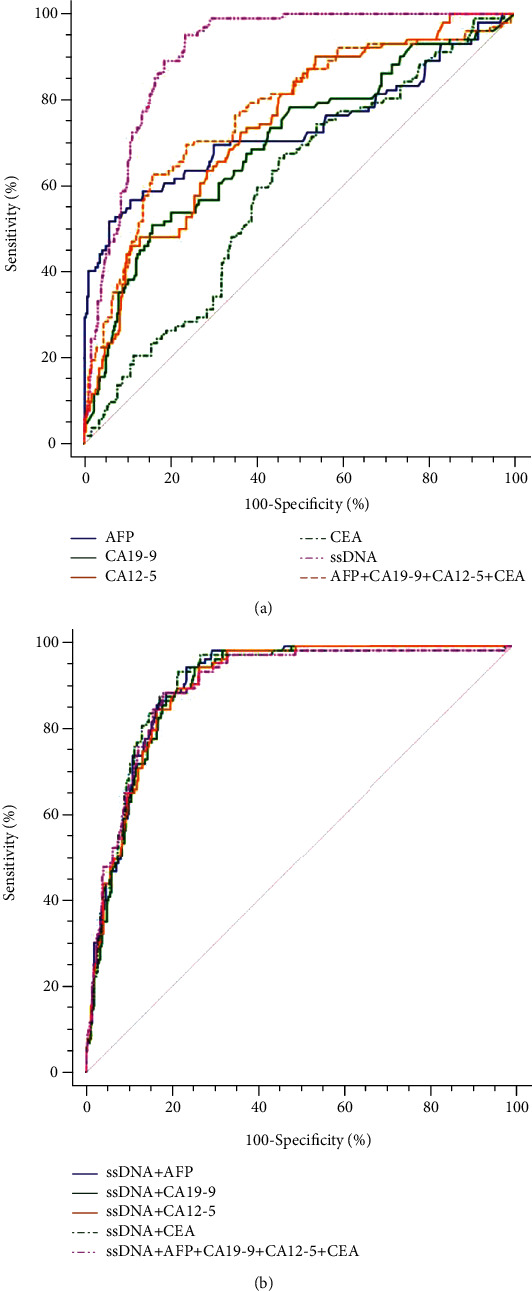
Receiver operating characteristic (ROC) curve of different markers for HCC diagnosis: *α*-fetoprotein (AFP), cancer antigen (CA) 19-9, CA12-5, carcinoembryonic antigen (CEA), and single-stranded DNA (ssDNA) in patients with hepatocellular carcinoma (HCC): (a) ROC curves of traditional tumor markers (TTMs) (AFP, CA19-9, CA12-5, and CEA) and ssDNA; (b) ROC of ssDNA combined with TTMs.

Table 3 and Figure 3(a) present the diagnostic values of ssDNA in patients with HCC (*n* = 102) and non-HCC subjects (*n* = 370). The AUC of ssDNA alone for HCC was 0.909 (95% CI: 0.879-0.933), with 95.1% sensitivity and 76.5% specificity (Table 3). The diagnostic efficiency and positive rates of ssDNA levels were similar between HCC with AFP < 20 ng/mL and AFP > 20 ng/mL and between cirrhosis-negative and cirrhosis-positive HCC (Supplementary Table S1 and Supplementary Figure S2). All combinations of ssDNA with the other TTMs did not significantly improve the diagnosis value of ssDNA alone for HCC (Table 3 and Figure 3(b)).

### 3.5. Diagnostic Value of TTMs in Patients with HCC

Table 3 presents the diagnostic values of the TTMs in patients with HCC (*n* = 102) and in non-HCC subjects (*n* = 370). The AUC of CA12-5 alone for HCC was 0.742 (95% CI: 0.700-0.781), with 90.2% sensitivity and 46.2% specificity. The AUC of AFP alone for HCC was 0.733 (95% CI: 0.691-0.722), with 56.9% sensitivity and 89.2% specificity. The AUCs of CA19-9 and CEA alone for HCC were all lower than those for CA12-5 and AFP. The sensitivity of combined TTMs (AFP, CA19-9, CA12-5, and CEA) was 62.8%, with 84.1% specificity, and the AUC was 0.772 (95% CI: 0.732-0.809). Multiple combinations were tested; all combinations that included ssDNA had higher AUCs than the combinations not including ssDNA.

### 3.6. Comparison of the Positive Rates of ssDNA among Different Diseases

Using ssDNA > 12.36 ng/*μ*L as the optimal cut-off value, the positive rate of ssDNA in patients with HCC (97/102) was significantly higher than that in the non-HCC subjects (*P* < 0.001) (Figure 2).

### 3.7. Changes in ssDNA Levels Pre- and Postradical Surgery in Patients with HCC

The paired ssDNA results showed that the ssDNA levels of 62 patients with resectable HCC peaked at 3 days after surgery and declined along with the decline of AFP levels (Supplementary Figure S3). The levels of ssDNA were stable around 60 days after surgery, and the levels decreased by 3.19-fold on average (*P* < 0.001) compared with baseline. According to the optimal cut-off values of ssDNA (>12.36 ng/*μ*L), the ssDNA levels of 11 patients with resectable HCC did not return to low levels (i.e., <12.36 ng/*μ*L) at 60 days after surgery, and six of those 11 patients were evaluated as CR based on RECIST.

### 3.8. Relationship between ssDNA Levels and Disease Prognosis

**Figure 4:**
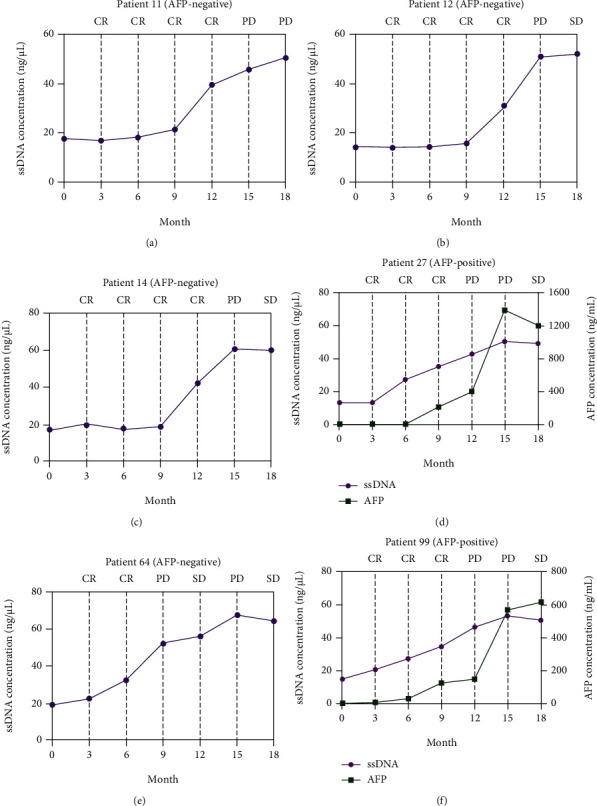
Line charts of single-stranded DNA (ssDNA) levels at serial time-points in patients with hepatocellular carcinoma (HCC) who achieved a complete response (CR) after radical surgery and confirmed with a recurrence during follow-up. SD: stable disease; PD: progressive disease. *α*-fetoprotein- (AFP-) positive HCC was defined as patients with AFP > 20 ng/mL.

A long-term follow-up of ssDNA levels in the six patients with CR mentioned above was performed to identify the prognosis value of ssDNA for HCC (Supplementary Table S2). The disease responses were evaluated according to RECIST 1.1. The line charts were plotted to describe the changes in ssDNA levels during follow-up. As shown in Figure 4, the ssDNA levels of the six patients increased at 6-12 months of follow-up but before disease progression was determined by RECIST.

### 3.9. Differences in ssDNA and dsDNA Levels according to the Extraction Method

Supplementary Figure S4 shows that the magnetic bead method yielded a better extraction efficiency than the QIAmp Circulating Nucleic Acid kit (Qiagen, Venlo, The Netherlands) (*P* < 0.01), while there were no differences for dsDNA between the two methods. In addition, the AUCs of ssDNA and dsDNA for the diagnosis of HCC were 0.909 and 0.880, respectively, with 95.1% and 88.2% sensitivity and 76.5% and 79.7% specificity.

## 4. Discussion

There is a lack of reliable biomarkers for HCC [7–9]. High plasma levels of ssDNA are a marker of genomic instability. This exploratory study is aimed at exploring the value of ssDNA as a marker of HCC diagnosis and prognosis by analyzing 102 patients with HCC and 370 controls. The results suggest that ssDNA might be a noninvasive indicator for HCC diagnosis and prognosis and might complement AFP and imaging, but studies are needed to confirm and validate the results. Still, the results suggest that ssDNA could be used to screen for HCC in patients with suspected HCC but negative AFP and inconclusive imaging.

The use of cfDNA in tumor diagnosis, treatment, and prognosis is promising [16–20]. The magnetic bead extraction of cfDNA yields high efficiency and quality [19–27, 32, 33, 35]. Therefore, in this study, magnetic beads were used to extract cfDNA. The Qubit ssDNA Assay Kit is not specific for ssDNA, and it will also detect dsDNA and RNA. Therefore, a methodological evaluation was performed to verify the extraction method of cfDNA. The results suggest that the ssDNA extraction efficiency using magnetic beads could be about twice that of the QIAmp Circulating Nucleic Acid kit (Qiagen, Venlo, The Netherlands), and the extracted nucleic acid was confirmed to be DNA. Since the ssDNA levels in cfDNA are much higher than dsDNA [23, 24], the interference of dsDNA on ssDNA determination might not affect the conclusions, but this will have to be validated in a specific methodological study.

The blood levels of cfDNA increase significantly when a patient suffers from tumors, autoimmune diseases, infectious diseases, stroke, and myocardial infarction [36]. In order to exclude the influence of those diseases on the diagnostic value of ssDNA levels for HCC, healthy individuals and patients with cirrhosis, chronic hepatitis, metabolic diseases, circulatory system diseases, autoimmune diseases, and various tumors other than HCC were used as various control groups. The results showed that the ssDNA levels were significantly higher in patients with HCC than all other diseases, and the ROC curve had an AUC of 0.909 for HCC diagnosis. Therefore, ssDNA seems to be a biomarker specific to HCC without interference from other diseases. Compared with AFP and TTMs, ssDNA might improve HCC diagnosis. The combination of ssDNA with any TTMs did not effectively improve the diagnostic value, suggesting that ssDNA might play a crucial diagnostic role for the diagnosis of HCC, as supported by a previous study [37]. Furthermore, the ssDNA levels in HCC were higher than those in chronic conditions associated with a higher risk of HCC (cirrhosis and chronic hepatitis) [7]. On the other hand, there were no differences in the diagnostic value among HCC patients with vs. without cirrhosis or with vs. without hepatitis, indicating that ssDNA is specific to HCC and has no interferences from concomitant liver conditions. Although replication stress is important but controllable in cirrhosis and chronic hepatitis, HCC has higher replication stress. Those results suggest that ssDNA had a good differential diagnosis effect on HCC and that it is possibly independent of cirrhosis and hepatitis. Indirectly supporting the present study, Kim et al. [38] showed that the expression of the ssDNA-binding protein 2 was elevated in patients with aggressive HCC. Du et al. [39] and Dong et al. [40] showed that aptamers specific to ssDNA could identify HCC. Nevertheless, the ssDNA cut-off value will have to be determined using large-scale multicenter studies. Indeed, the optimal cut-off ssDNA value in this study was >12.36 ng/*μ*L, while Chen et al. reported an optimal value of >509.98 ng/mL [41].

Interestingly, same as for cirrhosis-associated HCC, there were no differences in ssDNA levels, ssDNA diagnosis efficiency, and ssDNA positive rates between HCC with AFP < 20 ng/mL vs. >20 ng/mL. These results imply that ssDNA levels are possibly not related to AFP expression and that ssDNA could make up for the deficiency of AFP in the diagnosis of AFP-negative HCC.

ssDNA might be of use for the follow-up of patients. This study showed that, unlike AFP levels, the ssDNA levels began to decline after reaching a peak 3 days after surgery, which might reflect a rapid release of circulating tumor DNA after resection [42]. It is well known that the decline in postoperative AFP levels usually reflects the effectiveness of HCC treatment [43, 44]. In the present study, the ssDNA levels were stable over 60 days after surgery. When analyzing six patients with a complete response and whose ssDNA levels did not return to low levels (i.e., <12.36 ng/*μ*L) after surgery, ssDNA peaked at 6-12 months of follow-up, which was later followed by a confirmation of HCC recurrence by imaging. These results suggest that ssDNA might be used to indicate the effectiveness of HCC radical resection and for HCC prognosis before AFP and imaging. It is supported by Kim et al. [38], who showed that the levels of the ssDNA-binding protein 2 were associated with survival to HCC. Additional studies are necessary to confirm those results.

There were several limitations to the present study. First, since no data were available from the literature when this study was performed, a convenience sample of the patients who met the eligibility criteria during the study period had to be used. Second, the thresholds for the cut-off values were based on ROC analyses and need to be validated in an independent validation cohort. Third, given the modest number of HCC patients included in this study, the conclusion should be viewed with caution, especially in the presence of marginal *P* values. As this was an exploratory analysis, the subjects for the ROC analysis were simply grouped as HCC and non-HCC. Formal comparisons among different types of cancers and their characteristics will be the focus of future studies. Fourth, due to the small number of patients with follow-up, the applications of ssDNA in HCC progression and prognosis need further exploration. Fifth, the relationships between ssDNA and the efficacy of other HCC-related treatments have not been evaluated. Lastly, the ssDNA levels of unresectable HCC patients were higher than that of resectable HCC patients, but the present study did not explore whether ssDNA could be used to determine resectability. These issues still need further study.

## 5. Conclusions

This study suggests that ssDNA might be a noninvasive indicator for HCC diagnosis and prognosis. Confirmation of the results is necessary and the determination of the ssDNA cut-off value for HCC diagnosis.

## Figures and Tables

**Table 1:** Relationship between ssDNA levels and clinical characteristics of 102 patients with HCC.

Characteristics	*n* (%)	ssDNA levels (ng/*μ*L), median (IQR)	*P*
All	102 (100)	23.20 (16.86-44.40)	
*Age (years)*			
≤60	33 (32.4)	21.20 (15.75-58.60)	
60+	69 (67.6)	23.80 (18.05-39.84)	0.99^a^
*Sex*			
Male	77 (75.5)	23.80 (17.30-51.69)	
Female	25 (24.5)	21.20 (15.75-37.87)	0.42^a^
*AFP level*			
AFP-negative HCC	61 (59.8)	20.20 (15.88-50.33)	
AFP > 20 ng/mL	41 (40.2)	31.50 (18.69-37.58)	0.33^a^
*Cirrhosis*			
Cirrhosis-negative HCC	47 (46.1)	26.64 (16.34-52.35)	
Cirrhosis-positive HCC	55 (43.9)	21.46 (18.02-40.91)	0.64^a^
*Tumor size (mm) (sum of all tumors)*			
≤20	30 (29.4)	19.60 (15.20-48.60)	
20-50	35 (34.3)	19.20 (15.83-47.55)	
≥50	37 (36.3)	30.80 (20.07-38.39)	0.13^b^
*Tumor stage (TNM)*			
I	37 (36.3)	18.93 (15.98-34.70)	
II	42 (41.2)	22.63 (16.86-49.80)	
III-IV	23 (22.6)	34.80 (22.25-54.58)	0.02^b^
*Resected HCC*			
Yes	62 (60.8)	19.70 (15.80-37.80)	
No	40 (39.2)	28.50 (19.34-54.05)	0.02^a^
*Lymph node metastasis*			
Yes	13 (12.8)	48.60 (19.65-60.60)	
No	89 (87.2)	21.20 (16.85-38.39)	0.09^a^
*Distant metastasis*			
Yes	9 (8.8)	53.20 (25.85-96.00)	
No	93 (91.2)	21.20 (16.65-39.84)	0.04^a^
*HBV infection*			
HBV+	76 (74.5)	21.00 (16.49-44.40)	
HBV-	26 (25.5)	32.70 (18.18-48.60)	0.36^a^

Note: IQR: interquartile range. ^a^Comparison between the two groups was analyzed using the Mann–Whitney test; ^b^comparison among the multiple groups was analyzed using the Kruskal-Wallis test.

**Table 2:** Comparison of ssDNA levels between different liver diseases and healthy individuals.

Subjects	No.	ssDNA (ng/*μ*L), median (IQR)	*P*
Healthy individuals	45	9.36 (8.01-10.90)	
Cirrhosis	21	9.64 (8.93-11.68)	0.15^a^
Chronic hepatitis	20	9.76 (8.52-11.28)	0.39^a^
HCC	102	23.20 (16.86-44.40)	<0.001^b^

IQR: interquartile range. ^a^Comparison with healthy individuals was analyzed using the Mann–Whitney test; ^b^comparison among the multiple groups was analyzed using the Kruskal-Wallis test.

**Table 3:** Diagnostic efficiency of various variables for HCC.

Variable	Optimal cut-off value	SEN%	SPE%	AUC	95% CI	*P*
AFP (ng/mL)	>4.95	56.86	89.19	0.733	0.691-0.772	<0.001
CA19-9 (U/mL)	>38.8	50.98	84.05	0.700	0.656-0.741	<0.001
CA12-5 (U/mL)	>10.4	90.20	46.22	0.742	0.700-0.781	<0.001
CEA (ng/mL)	>2.57	66.67	54.59	0.595	0.549-0.640	0.002
AFP+CA19-9+CA12-5+CEA	>0.1763	62.75	84.05	0.772	0.732-0.809	<0.001
ssDNA (ng/*μ*L)	>12.36	95.10	76.49	0.909	0.879-0.933	<0.001
AFP+CA199	>0.1824	64.08	84.28	0.771	0.730-0.808	<0.001
AFP+CA125	>0.1891	66.99	74.53	0.771	0.730-0.808	<0.001
AFP+CEA	>0.2010	54.37	81.30	0.710	0.667-0.751	<0.001
CA199+CA125	>0	0.00	100.00	0.500	0.454-0.546	<0.001
CA199+CEA	>0.19279	71.84	61.52	0.705	0.662-0.746	<0.001
CA125+CEA	>0.20725	47.57	88.89	0.736	0.694-0.775	<0.001
AFP+CA199+CEA	>0.185	60.19	86.99	0.777	0.736-0.813	<0.001
AFP+CA199+CA125	>0.17613	66.02	82.11	0.783	0.743-0.819	<0.001
CA199+CA125+CEA	>0.18432	59.22	82.38	0.751	0.710-0.790	<0.001
ssDNA+AFP+CA199	>0.16065	87.38	81.30	0.902	0.872-0.927	<0.001
ssDNA+AFP+CA125	>0.1669	85.44	83.47	0.905	0.875-0.930	<0.001
ssDNA+AFP+CEA	>0.15144	93.20	78.05	0.907	0.877-0.932	<0.001
ssDNA+CA199+CA125	>0.16608	84.47	82.66	0.900	0.869-0.925	<0.001
ssDNA+CA199+CEA	>0.17043	85.44	83.47	0.900	0.869-0.925	<0.001
ssDNA+CA125+CEA	>0.17867	83.50	85.91	0.905	0.875-0.930	<0.001
ssDNA+AFP+CA199+CA125	>0.15393	89.32	78.86	0.902	0.872-0.927	<0.001
ssDNA+AFP+CA199+CEA	>0.16522	86.41	83.47	0.901	0.871-0.927	<0.001
ssDNA+CA199+CA125+CEA	>0.16153	87.38	81.57	0.901	0.870-0.926	<0.001
ssDNA+AFP	>0.1448	95.10	76.49	0.911	0.882-0.935	<0.001
ssDNA+CA19-9	>0.1433	95.10	74.59	0.904	0.874-0.929	<0.001
ssDNA+CA12-5	>0.1533	90.20	78.65	0.906	0.876-0.931	<0.001
ssDNA+CEA	>0.1529	94.12	78.38	0.909	0.880-0.934	<0.001
ssDNA+AFP+CA19-9+CA12-5+CEA	>0.1555	89.22	81.89	0.905	0.875-0.930	<0.001

AFP: alpha-fetoprotein; CA: cancer antigen; CEA: carcinoembryonic antigen; ssDNA: single-stranded DNA; SEN: sensitivity; SPE: specificity; AUC: area under the curve; CI: confidence interval.

## Data Availability

The datasets and data used and/or analyzed during the current study are available from the corresponding author on reasonable request.

## Supplementary Materials

Supplementary Materials Supplementary Table S1: ssDNA diagnostic efficiency for HCC. Supplementary Table S2. Marker characteristics of six patients with a complete response and available follow-up. Supplementary Figure S1. Relationship between ssDNA levels and tumor size (the sum of largest tumor diameters). (A) Linear relationship between ssDNA levels and tumor size. The equation was y=32.744+0.405x, r=0.17, P=0.095.The arrows in Supplementary Figure 1A point to the cases. (B) Case1 (tumor size: 11 mm; ssDNA level: 48.6 ng/*μ*L; AFP level: 267 ng/mL). (C) Case2 (tumor size: 84 mm; ssDNA level: 453 ng/mL; AFP level: 1.63 ng/mL) (D) Case3 (tumor size: 172 mm, ssDNA level: 23.8 ng/*μ*L, AFP level: 520 ng/mL). The arrows in Supplementary Figure 1B-D point to the lesions. Supplementary Figure S2. Comparison of the positive rates of ssDNA between AFP-negative HCC and AFP-positive HCC, and Cirrhosis-negative HCC and Cirrhosis-positive HCC. Supplementary Figure S3. Changes in ssDNA and AFP levels pre- and post-operation in HCC. (A) ssDNA. (B) AFP. ∗∗*P*<0.01. Supplementary Figure S4. Confirmation of cfDNA extraction efficiency of the magnetic bead method and cfDNA nature.(A) Comparison of ssDNA extraction efficiency between methods. (B) Comparison of dsDNA extraction efficiency between methods. (C) Agilent 2100 assay result for cfDNA extract before digestion. (D-E) Agilent 2100 assay results for cfDNA extract after RNase A digestion and DNase I digestion, respectively. NS: *P*>0.05. ∗∗*P*<0.01.
