# Supplementary material for: Role of ssDNA as a Noninvasive Indicator for the Diagnosis and Prognosis of Hepatocellular Carcinoma: An Exploratory Study
Source: Dis Markers. 2021 Aug 5;2021:9958909. doi: 10.1155/2021/9958909 (PMC8360730; doi:10.1155/2021/9958909)
Supplement: Supplementary Materials — Supplementary Table S1: ssDNA diagnostic efficiency for HCC. Supplementary Table S2. Marker characteristics of six patients with a complete response and available follow-up. Supplementary Figure S1. Relationship between ssDNA levels and tumor size (the sum of largest tumor diameters). (A) Linear relationship between ssDNA levels and tumor size. The equation was y=32.744+0.405x, r=0.17, P=0.095.The arrows in Supplementary Figure 1A point to the cases. (B) Case1 (tumor size: 11 mm; ssDNA level: 48.6 ng/μL; AFP level: 267 ng/mL). (C) Case2 (tumor size: 84 mm; ssDNA level: 453 ng/mL; AFP level: 1.63 ng/mL) (D) Case3 (tumor size: 172 mm, ssDNA level: 23.8 ng/μL, AFP level: 520 ng/mL). The arrows in Supplementary Figure 1B-D point to the lesions. Supplementary Figure S2. Comparison of the positive rates of ssDNA between AFP-negative HCC and AFP-positive HCC, and Cirrhosis-negative HCC and Cirrhosis-positive HCC. Supplementary Figure S3. Changes in ssDNA and AFP levels pre- and post-operation in HCC. (A) ssDNA. (B) AFP. ∗∗P<0.01. Supplementary Figure S4. Confirmation of cfDNA extraction efficiency of the magnetic bead method and cfDNA nature.(A) Comparison of ssDNA extraction efficiency between methods. (B) Comparison of dsDNA extraction efficiency between methods. (C) Agilent 2100 assay result for cfDNA extract before digestion. (D-E) Agilent 2100 assay results for cfDNA extract after RNase A digestion and DNase I digestion, respectively. NS: P>0.05. ∗∗P<0.01. [file 9958909.f1.docx]

**Supplementary Material**

**Supplementary Table S1.**ssDNA diagnostic efficiency for HCC

| **Groups** | **Cutoff value (ng/μL)** | **SEN%** | **SPE%** | **AUC** | **95% CI** | ***P*** |
| --- | --- | --- | --- | --- | --- | --- |
| **HCC** | >12.36 | 95.10 | 76.49 | 0.909 | 0.879-0.933 | <0.0001 |
| **AFP-negative HCC** | >11.50 | 98.36 | 63.50 | 0.851 | 0.815-0.882 | <0.0001 |
| **AFP-positive HCC** | >13.98 | 97.56 | 72.62 | 0.876 | 0.842-0.904 | <0.0001 |
| **Cirrhosis-negative HCC** | >13.98 | 91.49 | 72.94 | 0.863 | 0.828-0.892 | <0.0001 |
| **Cirrhosis-positive HCC** | >12.36 | 98.18 | 68.82 | 0.857 | 0.822-0.887 | <0.0001 |

AFP: alpha-fetoprotein; ssDNA: single-stranded DNA; SEN: sensitivity; SPE: specificity; AUC: area under the curve; CI: confidence interval.

**Supplementary Table S2.** Marker characteristics of six patients with a complete response and available follow-up

| **Patient** | ssDNA level (ng/μL) | | AFP level (ng/mL) | | RECIST  Evaluation |
| --- | --- | --- | --- | --- | --- |
|  | Pre-operation | Post-operation | Pre-operation | Post-operation |  |
| 11 | 51.92 | 17.51 | 1.62 | 1.85 | CR |
| 12 | 48.64 | 14.03 | 1.81 | 1.75 | CR |
| 14 | 55.83 | 17.01 | 2.57 | 2.06 | CR |
| 27 | 35.35 | 13.17 | 54000 | 7.86 | CR |
| 64 | 275.00 | 20.62 | 3.52 | 2.79 | CR |
| 99 | 37.50 | 14.92 | 54000 | 6.57 | CR |

AFP: alpha-fetoprotein; ssDNA: single-stranded DNA.

**
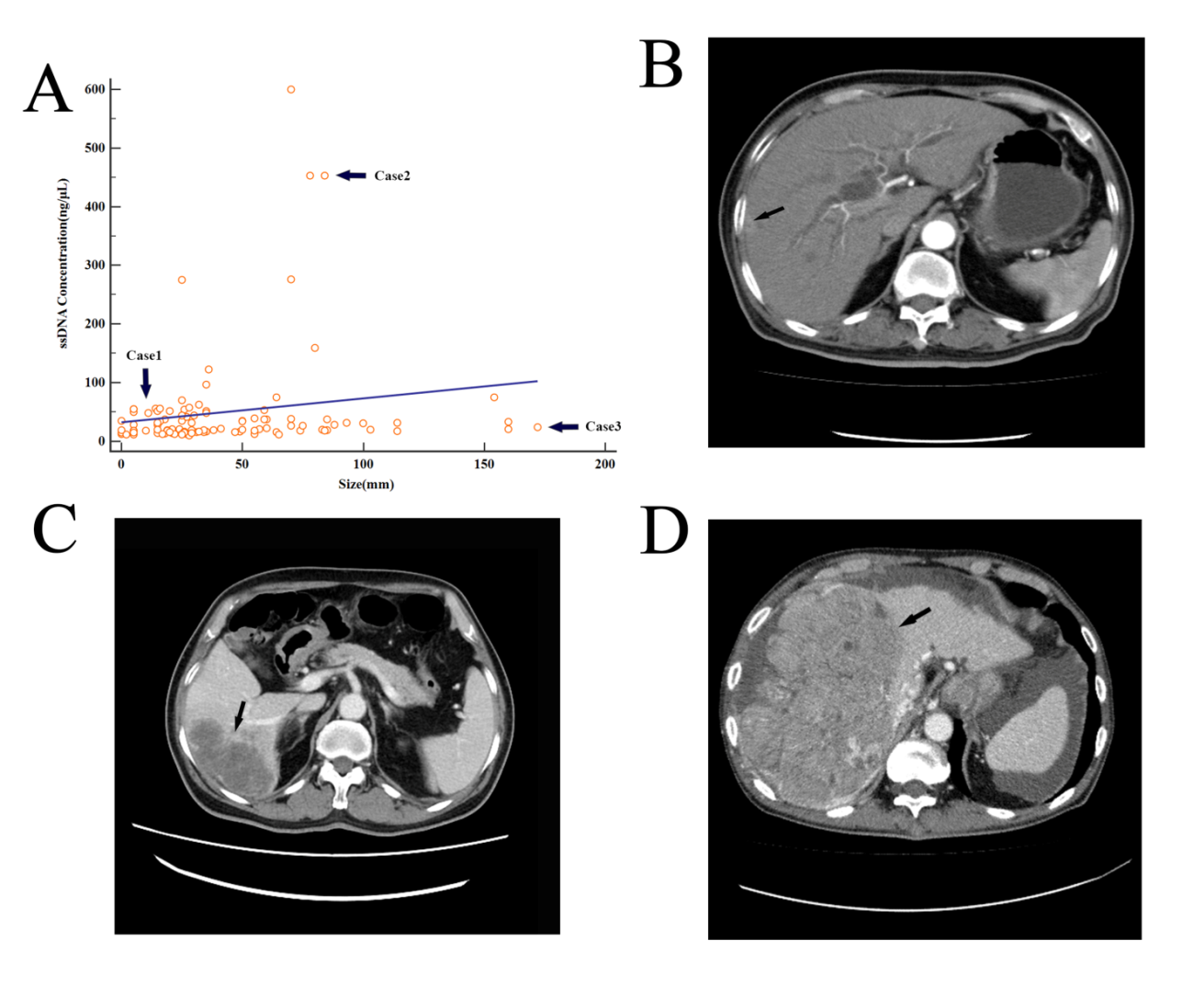
Supplementary Figure S1.** Relationship between ssDNA levels and tumor size (the sum of largest tumor diameters). (A) Linear relationship between ssDNA levels and tumor size. The equation was y=32.744+0.405x, r=0.17, P=0.095.The arrows in Supplementary Figure 1A point to the cases. (B) Case1 (tumor size: 11 mm; ssDNA level: 48.6 ng/μL; AFP level: 267 ng/mL). (C) Case2 (tumor size: 84 mm; ssDNA level: 453 ng/mL; AFP level: 1.63 ng/mL) (D) Case3 (tumor size: 172 mm, ssDNA level: 23.8 ng/μL, AFP level: 520 ng/mL). The arrows in Supplementary Figure 1B-D point to the lesions.

**Supplementary Figure S2.** Comparison of the positive rates of ssDNA between AFP-negative HCC and AFP-positive HCC, and Cirrhosis-negative HCC and Cirrhosis-positive HCC.


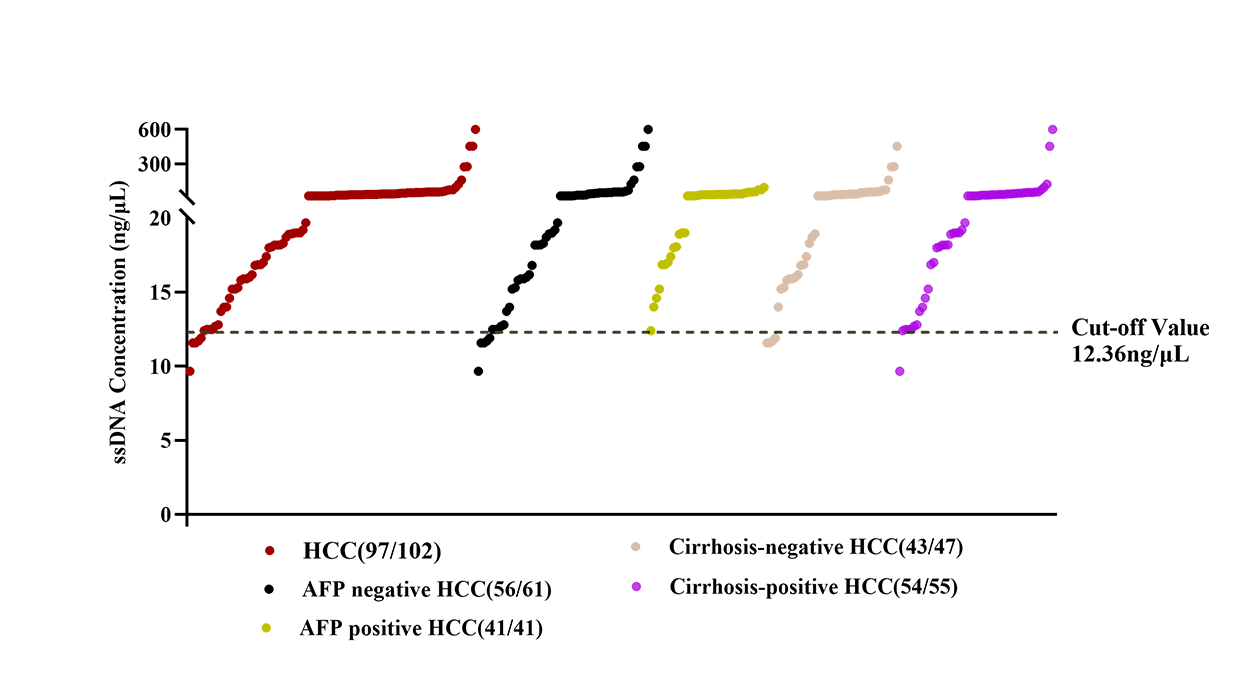


**Supplementary Figure S3.** Changes in ssDNA and AFP levels pre- and post-operation in HCC. (A) ssDNA. (B) AFP. ***P*<0.01.


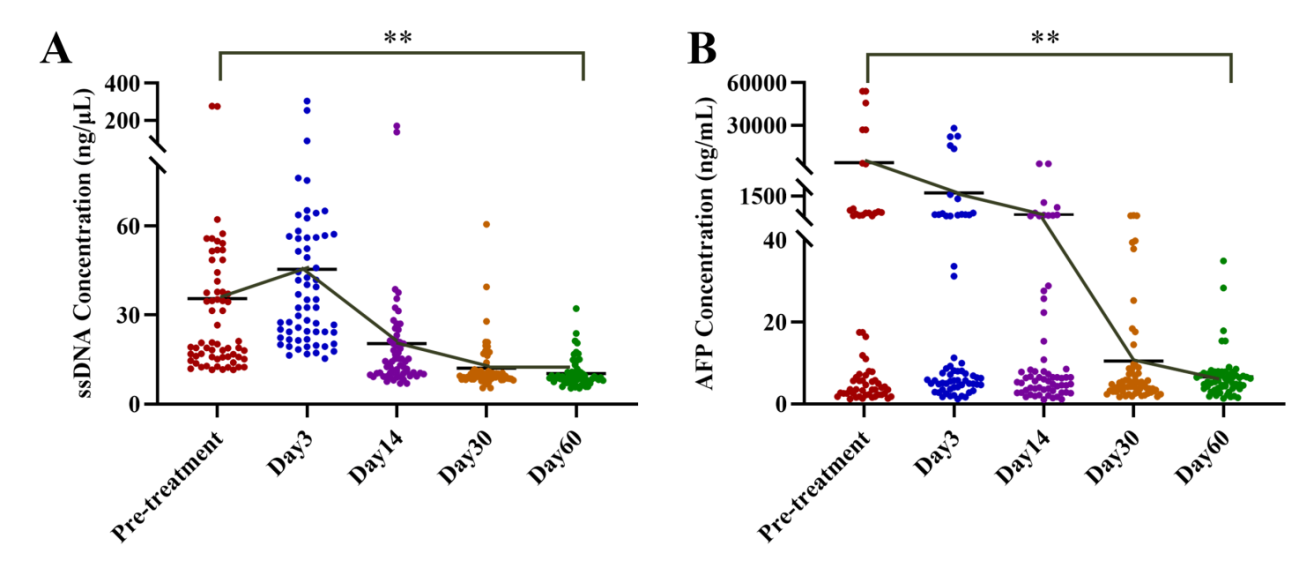


**
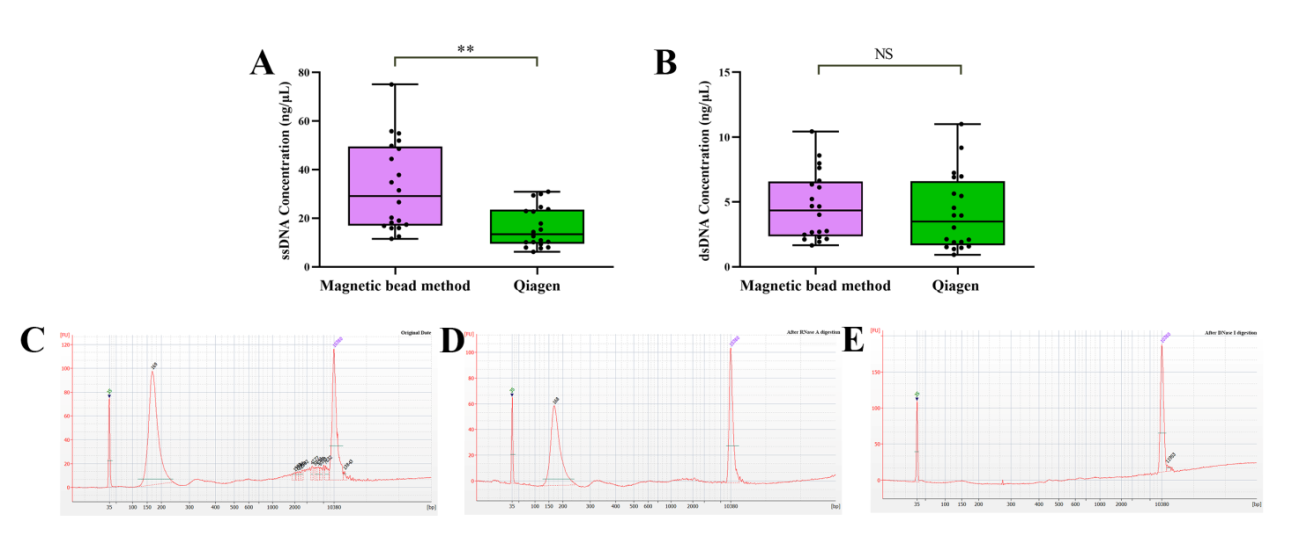
Supplementary Figure S4.** Confirmation of cfDNA extraction efficiency of the magnetic bead method and cfDNA nature.(A) Comparison of ssDNA extraction efficiency between methods. (B) Comparison of dsDNA extraction efficiency between methods. (C) Agilent 2100 assay result for cfDNA extract before digestion. (D-E) Agilent 2100 assay results for cfDNA extract after RNase A digestion and DNase I digestion, respectively. NS: *P*>0.05. ***P*<0.01.
